# Developing Relative Humidity and Temperature Corrections for Low-Cost Sensors Using Machine Learning

**DOI:** 10.3390/s21103338

**Published:** 2021-05-11

**Authors:** Ivan Vajs, Dejan Drajic, Nenad Gligoric, Ilija Radovanovic, Ivan Popovic

**Affiliations:** 1 Innovation Center, School of Electrical Engineering, University of Belgrade, Bulevar Kralja Aleksandra 73, 11120 Belgrade, Serbia; ddrajic@etf.bg.ac.rs (D.D.); ilija.radovanovic@ic.etf.bg.ac.rs (I.R.); 2 School of Electrical Engineering, University of Belgrade, Bulevar Kralja Aleksandra 73, 11120 Belgrade, Serbia; popovici@etf.bg.ac.rs; 3 DunavNET, DNET Labs, Trg Oslobodjenja 127, 21000 Novi Sad, Serbia; nenad.gligoric@dunavnet.eu; 4 Faculty of Information Technology, Alfa BK University, Palmira Toljatija 3, 11070 Novi Beograd, Serbia

**Keywords:** air pollution measurements, low-cost sensors, calibration, machine learning, artificial neural network, temperature and relative humidity

## Abstract

Existing government air quality monitoring networks consist of static measurement stations, which are highly reliable and accurately measure a wide range of air pollutants, but they are very large, expensive and require significant amounts of maintenance. As a promising solution, low-cost sensors are being introduced as complementary, air quality monitoring stations. These sensors are, however, not reliable due to the lower accuracy, short life cycle and corresponding calibration issues. Recent studies have shown that low-cost sensors are affected by relative humidity and temperature. In this paper, we explore methods to additionally improve the calibration algorithms with the aim to increase the measurement accuracy considering the impact of temperature and humidity on the readings, by using machine learning. A detailed comparative analysis of linear regression, artificial neural network and random forest algorithms are presented, analyzing their performance on the measurements of CO, NO_2_ and PM10 particles, with promising results and an achieved $R^{2}$ of 0.93–0.97, 0.82–0.94 and 0.73–0.89 dependent on the observed period of the year, respectively, for each pollutant. A comprehensive analysis and recommendations on how low-cost sensors could be used as complementary monitoring stations to the reference ones, to increase spatial and temporal measurement resolution, is provided.

## 1. Introduction

Most of the population is currently living in urban areas and a decade ago it was estimated that, at that time, the number was already higher than fifty percent [1], and the newest predictions published by WHO (World Health Organization) estimate that this number will increase up to seventy percent by the year 2050 [2]. Although the increase in population is not directly linked to the increase in pollution, a large number of people does give rise to a various number of pollution emitters. This is consequently accompanied by the increasing number of areas where the air pollution level is high above the defined ranges and could seriously affect the citizens’ health [3], which is associated with a series of acute and chronic diseases and is considered as one of the major health challenges at the moment (the limits for very high air pollutions: 50 $\frac{mg}{m^{3}}$ for CO, 400 $\frac{\mu g}{m^{3}}$ for NO_2_ and 180 $\frac{\mu g}{m^{3}}$ for PM10). In [4], it is reported that in the year 2016, in low and middle-income countries, the citizens’ mortality was heavily influenced by air pollution, and air pollution was linked to more than 4.2 million deaths per year (which represents 11.6% of all deaths). To combat that problem, the WHO issued the Air Quality Guidelines [5] about the recommendation regarding the activities concerning the pollution problem. There are also EU Directives defined on the ambient air quality [6,7] and many countries developed and implemented appropriate legislation. The most recognized air pollutants are CO, NO_2_, SO_2_, O_3_ and particulate matter (PM2.5, PM10). The EU Directive on reference methods, data validation and location of sampling points for the assessment of ambient air quality [7], contains detailed instructions and recommendations concerning the used reference methods, obtained data validation and selection of the location of sampling points for adequate air quality monitoring.

So far, in urban areas, the usual approach of the measurement of the air quality is the deployment of national networks of public monitoring stations, which are quite reliable, but, on the other hand, they are located at fixed positions, quite large and heavy [8]. Furthermore, they have a high price and annual recalibration costs, while due to the fixed and sparse positions, they provide the information only about the regional air quality while lacking the spatial resolution to provide local measurements, thus making the citizens’ exposure to the pollutants untrackable.

Contemporary, new generation, low-cost, off-the-shelf sensors look like a promising solution that could be used for complementary measurements for the areas that are not, and could not be, covered by public monitoring stations. Due to their high availability, low-cost sensors have great potential to be integrated into the portable low-cost Micro Sensing Units (MSUs) that can be used for air quality measurements. MSUs are mobile, have a wireless communication module and their maintenance costs are low. By applying the Internet of Things (IoT) concept, the data are remotely and periodically in real-time sent to a server in a cloud via the appropriate communication type (2G, 3G, 4G, WiFi, LoRa, etc.) where appropriate data storage, processing and visualization are performed [9]. They could be installed across the cities utilizing the existing public infrastructure (installed on public transport vehicles, public buildings, mounted on lamp posts, etc.). Additionally, it could be carried around by individuals, i.e., pedestrians and cyclists, thus allowing crowdsourcing [10], or even attached to drones. On the other hand, their main drawbacks are a short life cycle, low accuracy and most importantly, various influential calibration factors. The collected data might not always be accurate enough (due to the nature of electrochemical processes in the sensors and the influence of relative humidity, temperature and dust on the measurements) and in-field or laboratory calibration and periodical recalibrations are necessary, while the wireless transmission, in its nature, may introduce transmission errors and in the case of a wireless network failure, could be out of use. Furthermore, every sensor should be additionally calibrated, and the measurement accuracy of every single sensor highly depends on the sensor’s chemical and physical characteristics.

In the authors’ previous work [11], a methodology for the calibration of off-the-shelf air quality sensors is proposed and evaluated. The calibration process is based on the use of statistical algorithms and offset values obtained from the public measurement stations. The sensors were evaluated during a nine-month campaign in order to understand the seasonal influence on their behavior and a Common Air Quality Index (CAQI) [12] was calculated and compared with the public monitoring station. Obtained results were in a high level of agreement between the compared systems. The comparison between the results has shown that low-cost sensors could be used with a relatively high reliability as a complementary network to public monitoring stations, but it was also concluded that every sensor has its own sensitivity to temperature and relative humidity that influence the measurement accuracy.

Observed CO and NO_2_ sensors are electrochemical, and their performances are affected by temperature and relative humidity due to the nature of electrochemical processes ongoing during the measurements. Additionally, during the usage, the NO_2_ sensor has a higher loss of sensitivity than CO, and the NO_2_ gasis, by its nature, unstable at low concentrations. On the other hand, the influence of relative humidity and temperature on the PM10 sensor, which is optical, is caused by particle growth due to water absorption. The sensor sensitivity to temperature and relative humidity poses a great challenge, as it can hardly be modeled with a simple function. The linear regression (LR) model and the multi linear regression model (MLR), are the most widely used techniques to calibrate low-cost sensor data against a reference measurement. However, when modeling different dependencies is concerned, the scientific field of Artificial intelligence, more precisely machine learning (ML), has shown great promise. This field relies on different methods that have a basis in mathematical theory, and as such, have found many uses in both modern research and industry. Using the powerful tools of ML, it is possible to model a sensor’s dependencies on temperature and relative humidity and thus provide a more precise and reliable, yet low-cost measurement. In recent years, different types of Artificial Neural Networks (ANN) have been used for the calibration of low-cost air quality monitoring sensors in the laboratory or field conditions. Additionally, in order to achieve better results, for some low-cost air quality sensor types, it is recommended to examine the non-linear dependencies (exponential, logarithmic, quadratics) between the influencing variables, such as Random Forest (RF) [13,14], Support Vector Machines (SVR) [14,15] and the Gradient Boosting Regression Tree (GBRT) model [16]. The aim of this paper is to compare linear, different ANN and ML algorithms for in-field calibration of a low-cost sensor platform based on the collocation method.

### Related Work

The problem of field calibration methods for low-cost sensors was investigated in detail in [17,18]. The authors used the following calibration methods: LR, ANN and MLR. They have concluded that the most suitable calibration method was ANN using raw or scaled sensor inputs (higher correlation coefficient), while LR and MLR have been shown to produce lower performances, since these methods do not take into consideration all interfering factors with their weighted effect (relative humidity and temperature). For observed CO, CO_2_ and NO sensors, they concluded that ambient parameters such as relative humidity and temperature are necessary as algorithm inputs for appropriate calibration.

In [19], the authors stressed that the sensors’ performances are very sensitive to the environmental operating conditions, i.e., relative humidity and temperature due to the gas-sensing process that involves fairly complex reactions depending on the environment conditions, and that corresponding chemical reactions also vary from daytime to night-time in the urban atmosphere, which additionally degrades the performance of the sensors. They did not provide measurement principles, but rather discussed in detail the sensors and measurement devices issues with the focus on calibration issues. In general, manufacturers provide some correction factors for temperature and relative humidity, but for outdoor conditions, where relative humidity and temperature could change significantly on diurnal and seasonal bases, more sophisticated corrections are required.

In the scope of the CITY-SENSE project [20], authors tried to find the optimal calibration method for low-cost gas sensors for ambient air pollutants; the LR, MLR and ANN methods were compared and it was concluded that the ANN showed the best results for CO sensors.

In [21], data were collected from devices monitoring NO_2_, installed in traffic and the urban environment. A two-step calibration method was proposed; firstly, MLR was used, where the output is the value that contains the information about the error, which was then used as the input to more sophisticated algorithms: ANN, SVM and RF. The proposed method has shown that at high concentrations, NO_2_ sensors could closely meet the Air Quality Directive’s standards of accuracy, but they have also concluded that each individual sensor behaves differently. A very detailed analysis of the possibilities to correct the ambient PM measurement under high relative humidity (*RH*) conditions is presented in [22]. It was shown that by exploiting the measured particle size distribution, an adequate correction algorithm could be derived (using κ-Köhler theory) that highly improves measurement performance.

The authors [23] consider the problems concerning low-cost sensors calibration, having in mind the possible set of tens of thousands, or even millions, of air quality sensors deployed. They expect to use data storage and processing capability at the edge of the network [24]. For calibration, they propose the usage of a deep learning model consisting of convolution layers, fully connected neural network layers and long short-term memory (LSTM) layers that model temporal dependencies.

In [25], the authors investigated the performance for CO, NO_2_ and O_3_ sensors, first by using laboratory calibration, and then by conducting field experiments. They have performed the integration of ANNs with fuzzy logic, which leads to the creation of an adaptive neuro-fuzzy inference system (ANFIS) [26], thus making a single framework that uses the advantages of both techniques. The result evaluation shows that the ANFIS has high correlation coefficients in comparison to the reference system.

In [27], the authors explored the influence of relative humidity and the effect of atmospheric fog on the performance of a low-cost air particle mass PM sensor, in the laboratory and field conditions. The results have shown that there was no clear effect until relative humidity exceeded about 75%, while above this value, due to particle growth, the sensor started to show a steady increase in the measurements. The reason for this is that when the relative humidity is higher, it results in particle growth and fog that are detected by the particle monitoring equipment, that does not contain drying facilities at the sample inlets (which is the case with low-cost particle sensors). Observing this, it was concluded that this effect must be taken into account when using low-cost particle sensors in such environments.

The authors of [28] investigated the effect of relative humidity and air temperature on CO, NO, NO_2_ and O_3_. Tests were conducted for six relative humidity levels from 10% to 85% and four temperature levels of 10–45 °C in the laboratory. After the development of the correction algorithm, field measurements were performed (November 2019). A performance analysis showed that the developed algorithm improved the data quality of the sensors in most of the cases, as CO, NO, and NO_2_ sensors showed a satisfactory improvement, while the O_3_ sensor had the least improvement. When sensors were exposed to high temperatures, NO_2_ and O_3_ sensors mostly behaved poorly.

In [29], the authors used sensors from different manufacturers and performed a calibration by using different methods. They have concluded that for CO and NO sensors, the MLR methods were the best solution for calibration, although ANN shows the same performances as MLR for NO. For NO_2_ and O_3_ sensors, supervised learning models, such as SVR, RF and ANN, proved to be the best methods for calibration. For PM2.5, the best performances were obtained by using linear models, when the relative humidity measurements were less than 75%. For higher relative humidity values, the calibration using the Köhler theory is the most promising method.

In [30], an evaluation of the Aeroqual Ltd. Series 500 semiconducting metal oxide O_3_ and an electrochemical NO_2_ sensor was performed by comparison with UK national network reference analyzers for more than 2 months in central Edinburgh. The obtained O_3_ sensor measurements were in high correlation with the reference system, while the NO_2_ sensor suffered from co-sensitivity to O_3_, and the measurement error correction was developed by using LR.

A developed mobile PM2.5 sensing system was presented in [31], where eight sensing nodes were mounted on different city bus lines. Sensors were calibrated by using an ANN where the inputs, relative humidity and temperature were taken into account. A Gaussian Process regression algorithm was developed and implemented, so that by using measurements obtained from multiple sensors, PM2.5 values of locations within the observed region of interest, without direct measurements, could be interpolated.

In [32], an in-field measurement was conducted for CO, NO, NO_2_, O_3_, PM2.5, PM10 and SO_2_, and compared to the reference data. The calibration methods used were LR, ANN and RF. For the case of LR calibration, only the variable that was being calibrated was used as the input. For ANN and RF methods, all the measurements from each unit were used. In the case of CO, NO and NO_2_ sensors, satisfactory performances with LR were shown, but the additional improvement was obtained after the ANN and RF calibration. For the case of O_3_, ANN and especially RF calibration have shown better performances than LR. Finally, for the PM2.5, PM10 and SO_2_ sensors, both the ANN and the RF improve the results in comparison to the LR, and again, as in the previous case with O_3_, RF showed better performances than the ANN algorithm.

In [33], NO, NO_2_ and O_3_ were observed and the authors explored the performance of dynamic neural networks in comparison to the static feed-forward ANN, where relative humidity and temperature were taken into account. For all considered sensors, it was shown that the dynamic neural network architectures were superior to the classical feed-forward ANN, since its architecture considers several consecutive measurements, as opposed to the static ANN that considers only one. The design, implementation and evaluation of a novel client–cloud system are presented in [34], and two types of internet-connected particulate matter (PM2.5) monitors were created. Sensor calibration consisted of two algorithms that were combined, ANN and Gaussian Process regression. The main difference between the two algorithms was that the ANN was used for calibrating a single sensor, while the Gaussian Process regression was used to combine the data from multiple sensors with different confidence levels, which was proven in this paper to provide a significant improvement after the applied ANN calibration.

In [35], PM2.5 and PM10 were observed and three different algorithms were used for sensor calibration: LR, ANN and SVM. The algorithms were first implemented with two variants. Firstly, by using the PM concentration values, relative humidity and temperature as the inputs and the reference PM data as outputs. Secondly, the algorithms were implemented using the mentioned inputs with the addition of wind direction and wind speed. For each algorithm and particle type, the models performed better than in the first variant where wind direction and wind speed were not considered. With both input sets, the ANN was the superior algorithm.

In paper [36], authors performed a detailed study for the seasonal behavior of PM2.5, and applied different ML algorithms to perform sensor calibration, including temperature and humidity changes as factors that influence the accuracy of the sensors.

In Table 1, an overview of references used calibration methods, and commonly used metrics (correlation coefficient *R* and corresponding $R^{2}$ value, RMSE (Root Mean Squared Error) and NRMSE (Normalized Root Mean Squared Error) [29]) for evaluation are provided.

It should be noted that the authors used sensors from different manufacturers, device units from different manufacturers, different measurement sampling and averaging periods, different measurement campaign periods (total period of measurements and season) and different methodology (co-location method, laboratory method in controlled environment, mobile laboratory), so it is not possible to conduct a “fair” comparison of the metrics results.

The idea of the development and deployment of a low-cost sensor network for air quality monitoring is present in modern research. In [37] authors proposed a hybrid sensor network architecture with both stationary and mobile devices. They have developed a model for predicting the pollutant level, algorithms for hybrid network deployment and deployed a sensor network in a building. In [38] the capability of a network with low-cost PM sensors to capture PM spatial and temporal variations is explored. Six devices are mounted on fences/walls in the city of Southampton. The locations were chosen to be set around a school, while one of them was placed close to the road. Promising results were obtained, and in the next step, the authors plan to improve the spatial–temporal resolution by deploying 40 air quality monitoring devices in the area of 50 $km^{2}$ around the city. The authors of [39], deployed 24 air quality devices across the city of Oslo on the kindergarten premises. The focus was on measuring the NO_2_ (as one of the primary pollutants caused by traffic) to observe the proposed data fusion methodology for creating urban air quality maps. They showed that it was possible to obtain and extract valuable information from the deployed sensor network and develop urban air quality maps with high resolution by using the data fusion methodology. In [40], authors observed a network with 10 devices deployed in the city of Bari (schools, streets, port, buildings) on the fixed locations and one mobile device that was mounted on top of the public bus (CO, CO_2_, NO_2_, O_3_, SO_2_, PM1, PM2.5, PM10, *T* and *RH*). It was quite a long campaign (June 2015–December 2017) and after a detailed result analysis, it was concluded that the usage of low-cost sensor devices showed promising results that could address the data quality objective of the indicative measurements [6]. The authors of [41] developed a rapid deployment method for low-cost sensors deployment. The method has three phases: preparation, implementation and modification. In the first phase, the model is fed by basic input data (objectives, spatial data preparation, elimination rules), then the implementation phase includes information about the desired deployment density, unnecessary area elimination and algorithm settings. The proposed algorithm takes into account the geographic environment, available power supply, transmission networks, etc. The obtained result is the recommended number of sensor and deployment locations. In [42], the authors deployed 40 sensor devices (NO, NO_2_, CO, CO_2_) at the London Heathrow Airport and defined an analytical approach in order to distinguish long transport emissions from the airport emissions. The study was conducted during a five–week period (October–November 2012) and the implemented approach has managed to calculate ratios of the airport activities in different locations of the airport. They claim that their sensor network approach could be applied to a wide range of environmental pollution studies. A survey on existing state-of-the-art showed that the influence of *RH* and *T* on pollutant measurements is undisputable. It was also shown that different types of ML algorithms can successfully model these dependencies and improve the accuracy of various low-cost sensors. However, to the best of our knowledge, no paper has performed a comparative analysis of the calibration for low-cost sensors for CO, NO_2_ and PM10, taking into account *RH* and *T* influence, while comparing the results obtained with and without the *RH* and *T* as input features to the algorithm, thus quantifying the improvements *RH* and *T* can contribute to. Furthermore, no research paper has performed the calibration of low-cost CO, NO_2_ and PM10 sensors on data gathered from four different seasons, and tested the calibration of low-cost sensors using data from two consecutive years.

In this paper, the approach (LR calibration is used as a benchmark) from our earlier work [11] is taken further to additionally improve the calibration algorithms with the aim of increasing the measurement accuracy, taking into account the impact of the air *RH* and *T* on the readings by developing appropriate *RH* and *T* corrections by using ML. A detailed comparative analysis of the sensors’ behavior during a long observation time is performed (2 consecutive years). The selected observed months are from four different seasons (February, April, August, October), to ensure that the analysis of the applied ML algorithms performance is conducted on various weather conditions, thus taking into account different values of relative humidity and temperature depending on the observed season.

Even though the influence of *RH* and *T* on the low-cost sensors is “well-known”, and there is existing research that proves the correlation, there is no research that has quantified the differences in the performance of ML algorithms on calibration, including these two parameters (i.e., weather conditions). The calibration of a sensor was also conducted using a small sample of data from the observed month in combination with the data gathered from a preceding year.

The main contributions of the paper are the method and approach for the calibration of the low-cost sensors (CO, NO_2_ and PM10) using corrective measures (impact of *RH* and *T*), evaluated on different ML algorithms for the measurements taken during four different seasons over the period of two years. It was shown that all analyzed sensors are highly operable in the observed period (in accordance with their warranty period), with acceptable performances that are significantly improved by using proposed calibration algorithms and procedures, so that they can be used reliably in MSUs to provide a better spatial resolution within air quality measurement networks.

In addition to this, the discussion section contains a detailed analysis and recommendations on how low-cost sensors could be used for complementary measurements in order to increase spatial and temporal measurement resolution in combination with existing public monitoring networks. The deployment expenses are considered; the details about one possible low-cost monitoring station are provided from a practical point of view (device weight, dimensions, data transmission technology selection, etc.). Recommendations about the selection of location and mounting of a device are given. Finally, a hybrid sensor network approach is elaborated, which consists of reference monitoring stations supported by multiple low-cost devices. In this approach, low-cost sensors are virtually co-located with the reference monitoring station, thus making the recalibration process much easier. On the other hand, reference monitoring stations are supported and are implicitly expanded with spatially distributed complementary measurements.

The paper is organized as follows: In Section 2, the calibration procedure is explained, and the used ML methods are described. In Section 3, obtained results and the evaluation of performances are presented. In Section 4, a discussion about the results and paper contribution is elaborated. Finally, Section 5 provides conclusions and directions for future work.

## 2. Materials and Methods

### 2.1. Sensors and Data Collection

The collection of the data was performed by using a single low-cost sensor station and a single public air quality Automatic Monitoring Station run by the Serbian Environmental Protection Agency as a reference. The data from the public air quality monitoring station in Belgrade (Serbia) was collected during the period February–October during 2019 and in the same period (February–October) during 2020. The low-cost sensor station sensors are used from an air quality DunavNET ekoNET device AQ10x [9] for outdoor air quality measurements. This device is equipped with CO, NO_2_, SO_2_, O_3_ (Alphasense), temperature, air pressure, relative humidity sensors (Bosch BME 280), PM1, PM2.5 and PM10 (Plantower). The data from the device are then statistically correlated to the values captured from the official monitoring station for the exact same time intervals.

Having in mind that CO, NO_2_ and PM10 are not previously evaluated in this manner and that these are the most commonly used sensors, we have selected them for further evaluation. The reference measurement stations that were used in this paper provide pollutant measurements that are averaged on an hourly basis. On the other hand, the low-cost sensors that are used provide measurements every minute are then averaged for each hour to match the reference ones. Technical specifications of sensors are given in Table 2.

### 2.2. Calibration Methods

The performance of sensor devices (MSUs) is usually assessed using the mean error and/or correlation coefficients with respect to a reference laboratory or public monitoring stations’ equipment data. However, the behavior of the low-cost sensors calibrated in a laboratory can change from the laboratory to the field environment due to certain interferences (different gases, higher range of *T* and *RH*) that were not evaluated in the laboratory. In the field collocation of devices, with reference public monitoring stations or professional measuring instruments, measurements helped to compare and calibrate the low-cost sensors according to the data obtained, and in this case, the advantage is that the low-cost sensors were exposed directly to the desired environment in which they are to be deployed. Different approaches are used to increase the accuracy of the measurement and to develop correction algorithms. Although the low-cost sensors are to be tested under several established conditions and compared to reference instruments, there is a lack of uniform guidelines, protocols or standards for the application of this new technology for regulatory purposes [29].

For calibration purposes, one of the most common methods, (suitable also because of its implementation simplicity) the Least Squares Method (LSM) [43], was used. It performs line fitting based on the minimization of the sum of the squares of deviations from a straight line $S=\sum_{i=1}^{n}{\left(y_{i}-a-bx_{i}\right)}^{2}$ and calculates the line coefficients a and b. Let n be the number of experimental points, i.e., number of conducted measurements. Denoting by $y_{i}$ the reference values (from the public monitoring station) and by $x_{i}$ the measured values (from AQ10x device). After “calibration”, i.e., calculation of parameters a and b by LSM, the next step is to calculate the correlation of the obtained “calibrated” results with the results from the public monitoring station.

In Table 3, the mean, median and standard deviation values for *T* and *RH* for observed months and years are presented:

As a benchmark for a detailed study performed in this paper, in Table 4, corresponding $R^{2}$ coefficients are given for observed gases collected during four different parts of the year 2019 (February, April, August, October). LR calibration method is applied. For all four observed periods of interest, the sample size was a 15-day period, and the reference values are obtained once per hour (averaged measurement values per one hour), yielding the sample size of 15 × 24 = 360 per month.

From Table 4, it can be concluded that *T* and *RH* (stated in Table 1) considerably influence the behavior of low-cost sensors, which is visible for the period of February and August when low and high *T* influence measurements (the lowest $R^{2}$ was in August when temperatures were extremely high on average and in February when the temperatures were low). *RH* also had an influence, especially in the period when these values were high. Extreme values of *T* (low and high) and *RH* (high values) could cause a “peak” in the measurements from one side, and from the other, *T* (low and high) shifts the sensitivity of measurements to the lower levels, which correspondingly produces results with lower accuracy (it is visible in February and August).

### 2.3. Machine Learning Algorithms

**Figure 1:**
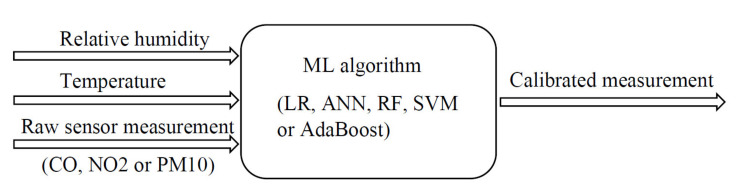
Measurement correction.

As the first step of calibration performance evaluation, several ML algorithms are selected that showed good performance in previous studies, and performed initial evaluation in order to obtain the most promising algorithms for further detailed evaluation. In this paper, a comparison between different ML algorithms using 10-fold cross-validation was performed with a 70/30 train–test split (for the data grouped together from all four observed periods). The evaluated algorithms were LR, two architectures of ANNs, RF, SVM and AdaBoost. The evaluation was performed for each measured pollutant separately, with the input for each algorithm being *RH*, *T* and the raw low-cost sensor data, and the output is the data from the reference sensor for the respective pollutant (Figure 1).

Each algorithm was evaluated using the metrics $R^{2}$, RMSE and NRMSE.

The results of the cross-validation are shown in Table 5.

The two algorithms that have achieved the best performance (highest $R^{2}$ and the lowest RMSE) regarding all three measured pollutants are ANN [44] (with 3 HL) and RF [45]. These two algorithms were used for further calibration testing.

During the initial cross-validation, two ANN architectures were tested, one with two hidden layers, and one with three hidden layers. Each of the hidden layers had 20 neurons, and the activation function of the hidden layers was the hyperbolic tangent. The ANN with three hidden layers had achieved better results for all pollutants, so this particular architecture was used for further calibration testing in this paper. The ANN overfitting was regulated by keeping the number of neurons per layer relatively low while tracking the loss function on the validation set (25% of the training set). The RF contained 100 decision trees and each decision tree had all three features (low-cost sensor measurement, *RH* and *T*) as the input since selecting anything less than three features would make some trees lack the low-cost sensor measurement as an input, which would make them unable to create valid predictions. Both the mentioned algorithms were implemented in the Python programming language. The RF was implemented using the scikit-learn library, while the ANN was implemented in TensorFlow.

## 3. Results and Performance Evaluation

In this section, obtained calibration results for the selected methods (LR, ANN and RF) are presented and the performance evaluation is conducted. Firstly, we observed the behavior of the selected algorithms when data from all four months in 2019 are concatenated. In Table 6. the averaged results of the cross-validation using data from all the months are presented. In the case of LR, there is no train (calibration)/test period, rather the algorithm is applied to the whole data set. For the RF and ANN algorithms, the results on the calibration set are expected to be better than the ones on the test set, but the test results correspond to the results that the algorithm could obtain in practice. Having this in mind, the ML algorithms will be compared based on the test set results, with the benchmark results being the ones obtained by the LR performed on the entire dataset.

It is shown that there is a clear difference between the results achieved when *RH* and *T* are included as the input to the ML algorithm calibration process. Better results were achieved regardless of which pollutant was selected, and regardless of the set type (calibration or test) in the case when *RH* and *T* are included as a calibration factor. The obtained results are to be expected since the influence of *RH* and *T* on low-cost sensors cannot be disputed. Furthermore, it is shown that both algorithms (RF and ANN) can model these influences successfully. It is also important to note that when the raw sensor data are the only input, ANN achieves superior results on the test set, regardless of the pollutant. This is most likely due to the ability of the ANN to better model non-linear functions of single variables due to the presence of activation functions. On the other hand, RF is superior if *RH* and *T* are taken into consideration.

It can be concluded that CO has the overall lowest value for NRMSE, which is expected, since CO generally shows the best $R^{2}$ value. It can also be observed that both the ANN and RF additionally lower the RMSE, and therefore the NRMSE value for each pollutant. By using the NRMSE parameters as a measure of comparison between the performances of the algorithms for different pollutants, we can see that the biggest improvement can be seen for the NO_2_ with the RF algorithm. This stands in line with the biggest improvement for the $R^{2}$ factor, which is present in the same case.

In the following text, we explore the calibration results for each observed month in 2019 separately. Table 7, Table 8, Table 9 and Table 10 contain the results obtained using the 10-fold cross-validation only on the data from the corresponding month in 2019 (i.e., February, April, August and October), with a 50/50 train/test split. This data split was used instead of the 70/30 one because of the size of the dataset for each individual month, to ensure testing was performed on a sufficiently large data sample. In the case of LR, there are no train/test periods, rather the algorithm is applied to the whole data set.

The results in Table 7 show that for the CO calibration, only the ANN algorithm surpassed the reference LR results. For the other two pollutants, RF has proven to be better with a significant improvement achieved for the NO_2_.

Results for the month of April stand in line with the results from February, indicating that the ANN models the CO sensor dependencies better than RF. Furthermore, PM10 and NO_2_ were better modeled by the RF, which is also in line with the results from the previous month.

The month of August has the lowest $R^{2}$ factor for the LR, for each pollutant. The improvements of this factor, however, are still present and indicate the applicability of the ML algorithms. In this month, the RF was shown to be better than the ANN for every pollutant.

The results from October show that the best algorithm for CO is the ANN. Regarding the NO_2_ and PM10 measurements, the RF was superior to the ANN.

It is shown that for every month in 2019, the RF obtained the best results both for NO_2_ and PM10 measurements. However, the results for the CO are mostly in favor of the ANN, which achieved the best results for every month except August, where the RF performed better. It is important to note that the trend of lowering the RMSE does correspond to the increase in the $R^{2}$ factor, in each observed month individually, and for every applied algorithm. The trends that the $R^{2}$ factor and RMSE follow within one month are important, but the comparison between months does have to include a careful evaluation since the lower concentrations of pollutants tend to influence the $R^{2}$ score negatively but can lower the RMSE.

**Figure 2:**
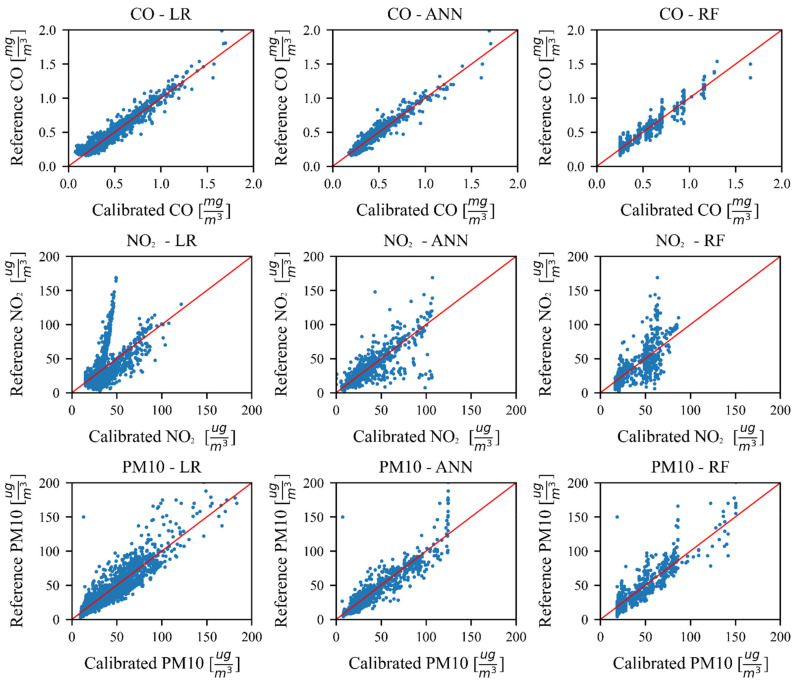
Test results from all observed months of 2019.

As a further step of evaluation, we present the scatter plots for different pollutants and the applied algorithms, i.e., LR, ANN and RF. For ANN and RF algorithms, the values from the test set are presented. In Figure 2, the results for the case where the data from all months in 2019 is concatenated together, are presented. The axis limits were chosen to maximize the usage of the space within each graph, and as such, cause a number of outlier measurements to be on the border of some graphs.

The scatter plots of the data from all months in 2019 show that if only the LR is implemented, the best correlation with the reference measurements is obtained for the CO. ANN and RF both improve the CO calibration, with the ANN having dispersed point placement and the RF having clusters. Particularly, the RF shows scatter points clustered into vertical lines. This means that for a small interval of reference measurements, the RF algorithm tends to return the same values. Although the NO_2_ low-cost sensor has the same measurement principle as the CO one, the nature of these pollutants and the sensors that measure them do vary. For example, in the NO_2_–LR scatter plot, it is clearly shown that by only using the raw sensor measurements as the inputs, a good linear correlation cannot be obtained, which was possible for CO. This is due to the nature of the data, as two different linear trends can be observed in the mentioned scatter plot. The ANN and RF algorithms show a clear improvement, although visibly less successful than the CO results. The PM10 scatter plots show that a single linear trend is present in the data and that both ML algorithms improve the correlation. It is interesting to note that due to the smaller number of data points (less than 50 in both the training and test set) with the higher PM10 concentration values (above 100 $\frac{ug}{m^{3}}$), the ANN seems to be unable to produce the higher values for PM10 concentration and maxes out at around 125 $\frac{ug}{m^{3}}$. The RF, on the other hand, does not seem to have this problem. The reason is due to the way both algorithms are structured, ANN has a single complex structure and adapts its weights numerically to optimize the loss function based on the data from the calibration set. Should the number of data points in a certain range be limited, their influence on the weights of the network will be insufficient to make the ANN output values in that particular range. On the other hand, the RF has many simpler structures (decision trees) where each is trained on a part of the calibration dataset, and this training process does not optimize a single model to the data, rather it fits many models on parts of the dataset.

In the following paragraphs, the results of the measurements obtained in the year 2020 are presented. The observation periods are the same as in 2019, i.e., for February, April, August and October. The methodology used for 2020 is the same as the one used for the year 2019, averaged hourly values obtained from devices were compared with measurements obtained from the reference station, for the same periods of the year on a 15-day level.

Firstly, we observe the $R^{2}$ values obtained by using LR on only the raw sensor data for the appropriate month in 2020. Secondly, we use all the data from 2019 as the training data and evaluate it on the data from a given month in 2020. Finally, we train a second RF on a sample of 4 days from the respective 2020 month and combine it with the RF trained on 2019 data. The idea is that by combining a small sample from the respective month with the data from the previous year, a significant improvement of the sensor performance could be achieved. The results obtained on the test sets (four different splits of 4/11 days of the respective 2020 month) were averaged and displayed in table format for each of the observation months of 2020. The RF algorithm was selected since it achieved the best results when using all the data from 2019 as shown in Table 5.

Observing the results obtained for the month of February 2020 (Table 11), using both the data from 2019 and 2020, the advantages of having a years’ worth of measurements are clear. Regarding the CO measurements, the results obtained after the calibration on the 2019 data decrease the $R^{2}$ factor, but also lower the RMSE. A similar result, with both the $R^{2}$ and RMSE lowered, is obtained using the RF trained on the four calibration days from 2020. Finally, the CO results obtained using a linear regression on the outputs of the two RF algorithms show a merely identical $R^{2}$ to the initial data, with the lowest RMSE out of all the previously mentioned cases. The NO_2_ measurements show that the linear combination of the RF algorithms shows the highest $R^{2}$ factor, followed closely by the 2019 RF algorithm. The linear combination of the RF algorithms achieves by far the lowest RMSE for the NO_2_ measurements. PM10 measurements show that the linear regression based on the outputs of two RF algorithms show the highest $R^{2}$ factor alongside the lowest RMSE, which stands in line with the data from the other two pollutants. Overall, for the month of February, combining the algorithms trained on the data from 2019 and 2020 gives the best results.

During the month of April (Table 12), there are some differences from the results obtained in February. In April, a state of emergency was declared in Serbia. This has, in turn, caused a steep decrease in the concentrations of all pollutants due to the lowered traffic. This made it more difficult for the algorithms to correctly pick up on the dependencies between the raw and reference data. The combination of two RF algorithms has a lower $R^{2}$ factor than both the raw data and the results from the 2019 RF calibration. On the other hand, the obtained RMSE for the linear combination of the RF algorithms is by far the lowest out of all the obtained results for the CO measurements. The NO_2_ results are similar to the results from February with the linear combination of the two RFs having both the highest $R^{2}$ and the lowest RMSE. The PM10 results show the highest $R^{2}$ factor for the raw data measurements. The results obtained from the RF calibrated on 2019 data are acceptable but the results from the 2020 calibration data are quite poor. This is due to the high variations of PM10 values in April 2020 (measurements up to 450 $\frac{\mu g}{m^{3}}$, while all other months’ measurements were up to 141 $\frac{\mu g}{m^{3}}$). This is quite interesting since the extremely high PM10 values (>200 $\frac{\mu g}{m^{3}}$) occurred after relaxing the state of emergency measures in Serbia. All other pollutant concentrations were also increased in the same period but not as drastically. The lowest RMSE is obtained for the linear combination of RFs but the $R^{2}$ factor is significantly decreased.

The results obtained for the month of August (Table 13) show a significantly lower $R^{2}$ value on the raw data for all pollutants, compared to the previous two observed months. The CO results show that the combination of RF algorithms based on data from 2019 and 2020 has the highest $R^{2}$ value and the lowest RMSE. The NO_2_ and PM10 measurements have a relatively low $R^{2}$ value on the raw data, but the RF algorithms behave differently for these two pollutants. The best results for the NO_2_ are obtained for the combination of the two RF algorithms, with the $R^{2}$ value almost unchanged from the raw data, but with a significantly lower RMSE. On the other hand, the PM10 results are quite poor indicating no possibility for calibration. The lifetime of a PM sensor based on the manufacturer declaration is 1 year and at the moment of these measurements, it was already 1 year and 7 months “old”, so this loss of accuracy is expected behavior. On the other hand, CO and NO_2_ sensors have a warranty of 2 years, but a slight degradation of accuracy is to be expected (notable for the NO_2_ sensor).

The results obtained from the October data (Table 14) indicate further degradation of the PM10 sensor and an operable state of the NO_2_ sensor. Although the results from August could indicate that both of the mentioned sensors suffered from significant degradation, it is clear that the NO_2_ sensor was still operable in October while the PM10 sensor has lost its functionality. The calibration results for CO show that the lowest RMSE was achieved when the two RF algorithms are combined, while the highest $R^{2}$ factor is present for the raw data, but with a significantly higher RMSE. For the NO_2_ results, the highest $R^{2}$ factor is obtained by combining both RF algorithms, while the lowest RMSE is obtained using only the RF trained on the data from 2019.

The results obtained from the data of 2020 show that a significant improvement in the sensors’ performance can be achieved by using a year’s worth of data in combination with just 4 days from a respective month. The CO sensor shows a high initial correlation for each month but an increased RMSE value when compared to the measurements from 2019, although the measurement value range was similar. This does imply sensor degradation, but the degradation can be easily modeled, and the results obtained from using both 2019 and 2020 data show promising results. The NO_2_ sensor does not achieve the results that are as good as the CO sensor, but it is still sufficiently accurate and shows an improvement with the implemented algorithms. The PM10 sensor has the most prominent degradation as it is practically unusable going forward from the month of August 2020 (while it is usable in February and April). Overall, apart from the limited lifetime of the PM10 sensor, the data acquired during 2019 has shown to be applicable in the calibration of the same sensor in 2020, with only 4 days from the observed month in 2020 as training data.

## 4. Discussion

In this paper, we have first considered data from CO, NO_2_ and PM10 obtained from a 9-month measurement campaign (from February to October 2019). In order to understand the behavior of the sensors’ performances, four different periods (February, April, August, October 2019) are observed, thus considering different values of *RH* and *T*. Different ML algorithms were used, that take into account *RH* and *T* in the calibration process, and the results are compared with the benchmark results obtained by the LR method. It was shown that the results from this experiment were satisfactory and that they can be further improved using the selected ML algorithms. This is important since it implies the possibility of using low-cost sensors alongside reference ones, to create better spatial and temporal measurement resolution. Generally, RF outperforms the ANN algorithm values except for the CO pollutant (although RF is better than the ANN in August). By using ML algorithms, the $R^{2}$ values are increased for all pollutants in the observed months. These improvements are summarized in Table 15.

The best improvement for every pollutant out of all the months in 2019 is achieved in the month of August (and after that in February, where the influence of *RH* and *T* on sensors was the second-highest). This could seem counter-intuitive since the best achieved $R^{2}$ values for August are the lowest out of all the months. However, the measurements of the pollutants in August show the lowest $R^{2}$ score when the LR algorithm is applied, indicating the high influence of weather conditions on the measurements in that month. The highest improvement rate achieved in August is a great example of how ML algorithms can achieve much more than a simple linear calibration, as they can successfully model non-linear dependencies between features. It is also important to mention that the achieved results for every individual month are obtained using cross-validation based only on the data from that particular month. The fact that such a clear improvement can be achieved with limited data acquisition represents a significant conclusion in this field of research. Acquiring air quality data is highly time-dependent as the process cannot be sped up in order to obtain a larger dataset. By showing that ML algorithms can be used both on every individual month, and on the concatenated data from all months, it is clear that ML algorithms do not only successfully scale up with larger datasets, but also that they can be scaled down to work with rather sparse data. Regarding the improvements for the pollutants, the highest $R^{2}$ increase for every month is achieved for NO_2_, followed by PM10, and finally CO. This could mean that the influence of *RH* and *T* on the low-cost sensors for NO_2_ is substantial and that the ML models successfully accommodated the sensors’ shortcomings. The CO correlation after LR is relatively high for each month, so a more modest improvement is expected, and PM10 particles stand somewhere in between CO and NO_2_ regarding the improvement rate.

In Table 16, the improvements when using data from all of the months are summarized. Both ML algorithms show improvements, but RF shows slightly better performances than ANN in all analyzed test cases, so only the improvements for RF are presented.

The improvements achieved using RF algorithms for the concatenated data from all of the months show that the ML algorithms can successfully be used on a dataset with varying weather conditions. It is also important to note that the results achieved for the concatenated data from all moths are obtained using a 70/30 train–test split, while the data for each individual month are obtained with a 50/50 train test–split. With a larger dataset and a more favorable train–test split, it would be expected that the improvements listed in Table 16 would be better than the individual improvements for each month, but that is not always the case. For example, the improvements for NO_2_ for the month of April are greater than the ones achieved for all months combined. The reason for this is the wide variety of values of *RH*, *T* and NO_2_ in the dataset consisting of all four months and a relatively low data count for such a feature space. If a substantial quantity of data were available, a deep learning algorithm could be implemented that would most probably successfully model all different dependencies. In this implementation, with a limited data quantity, the division of the calibration problem into monthly calibrations could be the optimal way, as is shown in the acquired results.

We have then focused on the measurement campaign conducted in the year 2020, repeating measurements with the same methodology as in the year 2019, the same four months are observed with the same measurement protocol. The observations from 2020 were used to analyze the possibility of using data from the preceding year to calibrate the same sensor in the present. It was also interesting to analyze the sensors’ performance after an entire year of in-field measurements.

The obtained values for the CO sensor show that the overall performance of the sensor in 2020 is quite equivalent to the one from 2019. Considering that the $R^{2}$ values are high for this sensor, a high usability of this low-cost device for at least two years is possible. The NO_2_ sensor does not have a performance as good as the CO one and the degradation is a bit more prominent. On the other hand, the $R^{2}$ factor during the 2020 months is still acceptable and shows that the NO_2_ sensor is also operable after two year’s worth of measurements. The PM10 sensor has shown to be the most sensitive and the results show it is operable through February and April 2020. This stands in line with the sensors’ warranties, as the CO and NO_2_ sensors have a 2-year warranty period and the PM10 sensor has a 1-year warranty.

The best-obtained results, using a combination of two RF algorithms, show a range of improvements. The improvements for the CO $R^{2}$ factor, ranging from 0.002 to 0.037, are overall not incredibly high. The initial $R^{2}$ for this pollutant is, however, quite high, and achieving a great improvement has shown to be unlikely. The NO_2_ $R^{2}$ factor has the best improvement out of all the considered pollutants, ranging from 0.001 to 0.12. The PM10 sensor has shown an improvement of 0.03 in February, where the calibration process could be applied. The obtained results do not differ greatly from the improvements that were achieved with the 2019 data.

In this paper, a comparative analysis of ML algorithms through a span of four months during two consecutive years (2019, 2020) is performed. The months selected are from four different seasons so that the analysis of the ML algorithm performance could be performed on various weather conditions. Furthermore, a comparative analysis between different ML algorithms was performed, as well as the investigation of the influence relative humidity and temperature can have on the calibration. The difference between the performance of algorithms that are based solely on the raw pollutant measurements, and the ones that include *RH* and *T* as input features are shown. An investigation of the possibilities of calibrating a sensor from the data gathered in the preceding year is also performed. It is shown that by combining the data from 2019 and a small sample of 4 days from the observed month in 2020, the improvements could be comparable to the results obtained in 2019 when 7.5 days from the observed month were used for calibration. This opens the possibility of reducing the duration of the calibration period of a low-cost sensor in a given month by using previously acquired data. It is important to note that different low-cost devices can perform differently and that one of the limitations of this work is that the analysis was performed on a single low-cost device. It was also impossible to acquire a continual stream of data from a reference monitoring station that could cover an entire year, which would surely be beneficial for the calibration process.

Based on this comprehensive study, it is proven that the measurement accuracy of every single sensor has its own sensitivity to *T*, *RH*, etc., and that for every pollutant a different approach for increasing the reliability of measurements should be developed and applied. By applying ML algorithms on the pollutant measurements, measurement accuracy is further improved, thus allowing low-cost sensors higher reliability and capability to be used as a complementary network to public monitoring stations, which will allow much higher measurement granularity, and the ability to observe air pollution at micro-locations. Furthermore, the integration of low-cost air quality measurement sensors will enable a higher density of air pollution assessment in urban areas and the development of sophisticated location-aware services for environmental protection, intelligent traffic control, accident detection, air pollutant transport and dispersion monitoring, etc. A detailed explanation of how it could be performed is provided in the following text.

### A Hybrid Sensors Network Approach

It is obvious that by increasing the number of deployed devices and providing a higher measurement frequency, one will obtain the results with better quality and accuracy, thus improving the detection of the sources of pollution and personal exposure. Low-cost devices are without a doubt more cost-effective than public monitoring stations. Based on the available vendor’s information, the average ratio is between 1:20 and 1:25, i.e., the cost of one public monitoring station is comparable with the cost of 20–25 low-cost devices for the same set of pollutants observed. In order to obtain more insight into the usage of one possible low-cost device [9], we have provided more detailed device characteristics and universal recommendations about the selection of the location and mounting of the device. Device dimensions are 180 × 180 × 265 ${\mathrm{mm}}^{3}$, weight is 1.5 kg and power consumption is 2.5 W. Different data transmission technologies are supported: GPRS, 3G, 4G, NB-IoT, LoRa, SigFox and WiFi. Generally, a low-cost device could be mounted on a wall, pole, pillar or some other solid object. It is also important to take into account the scope of monitoring (use case), distance from the pollution source, area topography, presence of different kinds of obstructions and the availability of appropriate deployment space. The objective of urban air quality monitoring is to capture and understand pollution trends and people exposure in the observed areas (depending on the use case it could be micro (up to 0.1 km), middle (0.1–0.5 km), neighborhood (0.5–4 km) or urban scales (4–50 km) [46,47]. Urban areas usually have local microclimate areas with different pollution conditions that could be of very small scales. Finally, in order to create a more accurate estimation of pollution, which is actually the goal of this paper, it is useful to install devices with low-cost sensors as complementary measurement devices that could be installed virtually anywhere. Collecting the data from these devices allows the creation of city pollution maps that can provide a deeper understanding of pollutants spatial distributions over specific areas, and on the other hand, high temporal resolution is provided using real-time measurements conducted every minute. In order to predict air quality with a higher accuracy, ML could be applied to help identify pollution hotspots. Reference monitoring stations are accurate but placed on fixed locations and quite expensive, while low-cost devices are cheap and mobile but suffer from a problem of accuracy and calibration. The most promising solution appears to be a combination of these two kinds of monitoring stations, i.e., the creation of a hybrid sensor network that combines the best of these two monitoring approaches. In this hybrid sensor network, a reference monitoring station is supported by multiple low-cost devices. In this way, sensors are virtually co-located with the reference monitoring station and their recalibration process is much easier (thus providing higher measurement accuracy), while reference monitoring stations are enhanced by spatially distributed complementary measurements. If some of the sensors start to suffer from in-accuracy, recalibration could be performed by correlation with a reference monitoring station or cross-calibration by comparison with recently re-calibrated devices in the area.Our future work will be devoted to the development of a model for the deployment of hybrid sensor networks and recommendations for the number of nodes and their spatial distribution (density).

## 5. Conclusions

In this paper, different ML algorithms are applied on the low-cost sensors’ measurements in order to improve the calibration algorithms taking into account the impact of the air *RH* and *T* on the readings.

The main contributions of the research described in this paper are the method and approach for the calibration of the low-cost sensors (CO, NO_2_ and PM10) using corrective measures (impact of *RH* and *T*). The method was evaluated on different ML algorithms for the measurements taken during four different seasons (February, April, August, October) in a period of two consecutive years.

The CO, NO_2_ and PM10, have shown satisfactory improvements after applying ML correction algorithms (the best improvements were obtained for NO_2_, then for PM10 and finally for CO). RF has shown better performances for NO_2_ and PM10 pollutants, while ANN was better for CO. With these corrections, the accuracy of the low-cost sensors’ measurement becomes more reliable and closer to the measurements obtained from reference monitoring stations. Depending on the observed period, $R^{2}$ is in the range from 0.927–0.970 for CO, 0.817–0.943 for NO_2_ and 0.731–0.891 for PM10.

After the analysis of the data from 2019, data from 2020 was taken into consideration. The 2020 data was gathered during the same months as the data from 2019 to observe sensor degradation and the possibility of calibration based on the data from the preceding year. The obtained results show that a valuable improvement on the sensors’ performance can be achieved by using 2019 data in combination with just 4 days from a respective month in 2020. Regarding sensor degradation, the results are promising for the CO and NO_2_ sensors, while the PM10 sensor had significant degradation in the second half of 2020.

Finally, the results of the research have shown that the low-cost sensors with adequate correction algorithms could be used as good support for the current traditional air quality monitoring stations. A detailed analysis performed on how low-cost sensors could be used for measurements in order to increase spatial and temporal measurement resolution together with public reference monitoring stations, i.e., a hybrid sensor network approach is elaborated.

For future work, the influence of weather conditions on other types of pollutant measurements using low-cost sensors (SO_2_, PM2.5, O_3_) will be performed. The cross-sensitivity between pollutants can also be measured, by experimenting with different pollutants as input features to the ML algorithms. The development of more complex ML models (1D convolutional neural networks and long short-term memory networks) will also be conducted, which will be trained on larger data samples. Finally, a hybrid sensor network approach will be analyzed in more detail. The possibilities of cross-calibration between low-cost sensors will be performed, by calibrating several low-cost sensors at the same measuring site and analyzing if the calibration models can be swapped between the sensors and still obtain satisfactory results.

## Figures and Tables

**Table 1:** Types of calibration models used in the literature.

Pollutant	Calibration Model	References	Metrics
CO	LR	Drajic [11], Spinelle [17], Spinelle [18], Topalovic [20], Samad [28], Karagulian [29], Lin [30], Borrego [32]	R, $R^{2}$, RMSE, NRMSE
CO	ANN	Spinelle [17], Spinelle [18], Topalovic [20], Motlagh [23], Alhasa [25], Karagulian [29], Borrego [32]	R, $R^{2}$, RMSE, NRMSE
CO	RF	Karagulian [29], Borrego [32]	$R^{2}$, RMSE
NO_2_	LR	Drajic [11], Spinelle [17], Spinelle [18], Cordero [21], Karagulian [29], Borrego [32]	$R^{2}$, RMSE
NO_2_	ANN	Spinelle [17], Spinelle [18]. Motlagh [23], Alhasa [25], Samad [28], Karagulian [29], Borrego [32], Espositi [33]	$R^{2}$, RMSE
NO_2_	RF	Cordero [21], Karagulian [29], Borrego [32]	$R^{2}$, RMSE
PM10	LR	Drajic [11], Jayaratne [27], Karagulian [29], Borrego [32]	$R^{2}$, RMSE
PM10	ANN	Motlagh [23], Karagulian [29], Borrego [32]	$R^{2}$, RMSE
PM10	RF	Karagulian [29], Borrego [32]	$R^{2}$, RMSE
PM2.5	LR	Di Antonio [22], Chen [35]	$R^{2}$, RMSE
PM2.5	ANN	Gao [31], Chang [34], Chen [35]	$R^{2}$, RMSE
PM2.5	RF	Wang [36]	$R^{2}$, RMSE

**Table 2:** Sensor’s characteristics.

Pollutant	Manufacturer	Model	Range	Unit
CO	Alphasense	CO-B4	0–50 ppm	ppm or mg/m^3^
NO_2_	Alphasense	NO_2_-B43F	0–20 ppm	ppb or μg/m^3^
PM10	Plantower	PMS7003	0~1000 μg/m^3^	μg/m^3^

**Table 3:** Averaged/Median/Standard deviation (Std) values for *T* and *RH*.

Parameter	February	April	August	October
Average T [°C] 2019 Average T [°C] 2020	6.8 7.7	9.2 11.7	25.1 23.7	16.3 18.6
Median T [°C] 2019 Median T [°C] 2020	8.1 5.9	11.1 9.7	23.2 24.9	17.9 16.1
Std T [°C] 2019 Std T [°C] 2020	5.5 3.9	4.9 5.7	4.6 4.5	4.5 3.9
Average *RH* [%] 2019 Average *RH* [%] 2020	74.1 71.3	54.3 48.9	59.2 60.1	64.9 62.1
Median *RH* [%] 2019 Median *RH* [%] 2020	70.9 72.7	51.1 52.1	61.3 59.5	61.8 64.1
Std *RH* [%] 2019 Std *RH* [%] 2020	16.5 17.4	16.1 17.1	19.3 15.1	16.4 15.8

**Table 4:** Coefficients obtained for observed periods of 2019.

Pollutant	$R^{2}$			
	February	April	August	October
CO	0.933	0.949	0.861	0.946
NO_2_	0.784	0.846	0.671	0.828
PM10	0.716	0.849	0.664	0.786

**Table 5:** Averaged metrics calculated on the test sets during cross-validation 2019.

Algorithm	CO		NO_2_		PM10	
	$R^{2}$	RMSE	$R^{2}$	RMSE	$R^{2}$	RMSE
Linear regression	0.935	0.066	0.737	13.412	0.837	12.551
Neural network 1 (2 HL ^1^)	0.941	0.065	0.869	9.450	0.839	12.583
Neural network 2 (3 HL)	0.943	0.063	0.872	9.344	0.850	12.124
AdaBoost	0.924	0.074	0.843	10.360	0.846	14.560
Random forest	0.945	0.060	0.894	8.540	0.872	11.123
SVM	0.933	0.070	NC ^2^	NC	0.835	12.748

^1^ HL, hidden layer; ^2^ NC, non-convergent.

**Table 6:** All months 2019, CO, NO_2_, PM10, LR, ANN, RF, calibration and test set.

Pollutant, Algorithm (Input Features)	$R^{2}$		RMSE		NRMSE
	Calibration	Test	Calibration	Test	Test
CO, LR (raw)	**0.931**		**0.068**		**0.264**
CO, ANN (raw)	0.927	0.927	0.070	0.070	
CO, ANN (raw, *RH*, *T*)	0.945	0.943	0.061	0.063	0.244
CO, RF (raw)	0.988	0.915	0.028	0.075	
CO, RF (raw, *RH*, *T*)	0.994	**0.945**	0.022	**0.060**	**0.233**
NO_2_, LR (raw)	**0.793**		**11.980**		**0.455**
NO_2_, ANN (raw)	0.809	0.797	11.610	11.913	
NO_2_, ANN (raw, *RH*, *T*)	0.908	0.872	8.040	9.340	0.348
NO_2_, RF (raw)	0.967	0.762	4.817	12.860	
NO_2_, RF (raw, *RH*, *T*)	0.986	**0.894**	3.162	**8.543**	**0.325**
PM10, LR (raw)	**0.794**		**14.112**		**0.453**
PM10, ANN (raw)	0.782	0.774	14.687	14.969	
PM10, ANN (raw, *RH*, *T*)	0.910	0.850	9.482	12.121	0.389
PM10, RF (raw)	0.959	0.709	6.374	17.198	
PM10, RF (raw, *RH*, *T*)	0.982	**0.872**	4.140	**11.124**	**0.357**

**Table 7:** February 2019, CO, NO_2_, PM10, LR, ANN, RF.

Pollutant, Algorithm (Input Features)	$R^{2}$		RMSE	
	Calibration	Test	Calibration	Test
CO, LR (raw)	**0.933**		**0.053**	
CO, ANN (raw, *RH*, *T*)	0.980	**0.968**	0.031	**0.038**
CO, RF (raw, *RH*, *T*)	0.993	0.934	0.017	0.052
NO_2_, LR (raw)	**0.784**		**8.940**	
NO_2_, ANN (raw, *RH*, *T*)	0.857	0.832	7.986	8.625
NO_2_, RF (raw, *RH*, *T*)	0.985	**0.904**	2.360	**5.976**
PM10, LR (raw)	**0.716**		**12.012**	
PM10, ANN (raw, *RH*, *T*)	0.780	0.737	11.567	12.549
PM10, RF (raw, *RH*, *T*)	0.962	**0.767**	4.436	**10.221**

**Table 8:** April 2019, CO, NO_2_, PM10, LR, ANN, RF.

Pollutant, Algorithm (Input Features)	$R^{2}$		RMSE	
	Calibration	Test	Calibration	Test
CO, LR (raw)	**0.949**		**0.054**	
CO, ANN (raw, *RH*, *T*)	0.982	**0.974**	0.032	**0.039**
CO, RF (raw, *RH*, *T*)	0.996	0.970	0.015	0.042
NO_2_, LR (raw)	**0.846**		**9.278**	
NO_2_, ANN (raw, *RH*, *T*)	0.889	0.866	9.463	10.001
NO_2_, RF (raw, *RH*, *T*)	0.993	**0.943**	2.008	**5.695**
PM10, LR (raw)	**0.849**		**8.070**	
PM10, ANN (raw, *RH*, *T*)	0.888	0.867	8.111	8.680
PM10, RF (raw, *RH*, *T*)	0.984	**0.891**	2.806	**7.204**

**Table 9:** August 2019, CO, NO_2_, PM10, LR, ANN, RF.

Pollutant, Algorithm (Input Features)	$R^{2}$		RMSE	
	Calibration	Test	Calibration	Test
CO, LR (raw)	**0.861**		**0.048**	
CO, ANN (raw, *RH*, *T*)	0.894	0.885	0.039	0.047
CO, RF (raw, *RH*, *T*)	0.978	**0.927**	0.019	**0.033**
NO_2_, LR (raw)	**0.671**		**11.286**	
NO_2_, ANN (raw, *RH*, *T*)	0.940	0.767	4.590	10.130
NO_2_, RF (raw, *RH*, *T*)	0.961	**0.817**	3.620	**9.460**
PM10, LR (raw)	**0.664**		**8.740**	
PM10, ANN (raw, *RH*, *T*)	0.813	0.678	6.985	8.664
PM10, RF (raw, *RH*, *T*)	0.967	**0.731**	2.882	**7.935**

**Table 10:** October 2019, CO, NO_2_, PM10, LR, ANN, RF.

Pollutant, Algorithm (Input Features)	$R^{2}$		RMSE	
	Calibration	Test	Calibration	Test
CO, LR (raw)	**0.946**		**0.068**	
CO, ANN (raw, *RH*, *T*)	0.969	**0.968**	0.052	**0.062**
CO, RF (raw, *RH*, *T*)	0.991	0.949	0.028	0.067
NO_2_, LR (raw)	**0.828**		**13.761**	
NO_2_, ANN (raw, *RH*, *T*)	0.893	0.875	10.880	11.820
NO_2_, RF (raw, *RH*, *T*)	0.988	**0.914**	3.698	**9.786**
PM10, LR (raw)	**0.786**		**16.492**	
PM10, ANN (raw, *RH*, *T*)	0.910	0.819	4.550	9.570
PM10, RF (raw, *RH*, *T*)	0.977	**0.824**	5.623	**8.940**

**Table 11:** February 2020 test results, CO, NO_2_, PM10.

Pollutant (Input Set)	$R^{2}$	RMSE
CO, LR (raw)	0.952	0.091
CO, RF (2019)	0.953	0.077
CO, RF (2019 + 2020)	0.957	0.065
NO_2_, LR (raw)	0.830	18.564
NO_2_, RF (2019)	0.853	15.667
NO_2_, RF (2019 + 2020)	0.856	10.564
PM10, LR (raw)	0.833	28.356
PM10, RF (2019)	0.844	12.071
PM10, RF (2019 + 2020)	0.863	11.046

**Table 12:** April 2020 test results, CO, NO_2_, PM10.

Pollutant (Calibration Set)	$R^{2}$	RMSE
CO, LR (raw)	0.954	0.079
CO, RF (2019)	0.955	0.064
CO, RF (2019 + 2020)	0.956	0.051
NO_2_, LR (raw)	0.569	23.625
NO_2_, RF (2019)	0.676	21.973
NO_2_, RF (2019 + 2020)	0.689	15.316
PM10, LR (raw)	0.786	71.302
PM10, RF (2019)	0.732	49.949
PM10, RF (2019 + 2020)	0.739	48.516

**Table 13:** August 2020 test results, CO, NO_2_, PM10.

Pollutant (Calibration Set)	$R^{2}$	RMSE
CO, LR (raw)	0.764	0.074
CO, RF (2019)	0.787	0.054
CO, RF (2019 + 2020)	0.801	0.035
NO_2_, LR (raw)	0.476	24.134
NO_2_, RF (2019)	0.440	17.834
NO_2_, RF (2019 + 2020)	0.477	7.917
PM10, LR (raw)	0.408	17.935
PM10, RF (2019)	0.303	8.872
PM10, RF (2019 + 2020)	0.249	8.201

**Table 14:** October 2020 test results, CO, NO_2_, PM10.

Pollutant (Calibration Set)	$R^{2}$	RMSE
CO, LR (raw)	0.901	0.081
CO, RF (2019)	0.903	0.069
CO, RF (2019 + 2020)	0.904	0.059
NO_2_, LR (raw)	0.748	15.432
NO_2_, RF (2019)	0.779	10.993
NO_2_, RF (2019 + 2020)	0.785	10.366
PM10, LR (raw)	0.213	30.217
PM10, RF (2019)	0.134	26.418
PM10, RF (2019 + 2020)	0.219	34.650

**Table 15:** $R^{2}$ improvements for CO, NO2, PM10, LR, ANN, RF, by months in 2019.

Pollutant	$R^{2}\;\mathrm{Improvement}$			
	February	April	August	October
CO	0.035	0.025	0.066	0.022
NO_2_	0.120	0.097	0.146	0.086
PM10	0.051	0.042	0.067	0.038

**Table 16:** $R^{2}$ improvements for CO, NO_2_, PM10, RF, all months in 2019.

Pollutant	$R^{2}\;\mathrm{Improvement}$
CO	0.014
NO_2_	0.101
PM10	0.078

## Data Availability

Not applicable.
